# Knowledge discovery of diseases symptoms and rehabilitation measures in Q&A communities

**DOI:** 10.1038/s41598-025-98300-9

**Published:** 2025-04-19

**Authors:** Yanli Zhang, Tao Wang, Yan Wang, Jingyu Cao

**Affiliations:** 1 College of Business Administration, Henan Finance University, Zhengzhou, 451464 China; 2 School of Politics and Law, Hubei University of Arts and Science, Xiangyang, 441053 China; 3 Global Cooperation Division, China Development Bank Qinghai Branch, Xining, 810001 China; 4 Information Technology Office, Henan Finance University, Zhengzhou, 451464 China

**Keywords:** Rehabilitation Q&A community, Relationship extraction, Rehabilitation knowledge mining, Gated recurrent unit, Attention mechanism, Computational biology and bioinformatics, Health care

## Abstract

Rehabilitation-related diseases have long recovery times, making frequent hospital visits impractical for patients. There is a high demand for online rehabilitation advice, but valuable Q&A information in online health communities remains largely untapped, leading to wasted medical resources. This study developed a BERT-BiGRU-attention model to extract three types of entity relationships: disease symptoms, appropriate rehabilitation measures, and inappropriate rehabilitation measures. This model achieved optimal knowledge extraction results. We then used a clustering analysis model to group disease-related knowledge, helping to uncover useful information for rehabilitation patients, assist in medical diagnosis, and enhance health education.

## Introduction

Medical Rehabilitation is a systematic project that helps patients restore physical function, psychological state, and social participation capabilities through multidimensional interventions^1^. The importance of medical rehabilitation has become increasingly prominent, yet the field currently faces two core challenges: (1) Fragmented Knowledge: Rehabilitation services require the integration of multidisciplinary knowledge (e.g., physical therapy, psychological interventions), but the lack of information-sharing mechanisms among institutions leads to uneven resource allocation and inconsistent service quality; (2) Lagging Standardization: Traditional clinical rehabilitation guidelines often rely on generalized protocols, failing to address individual patient needs (e.g., personalized interventions for elderly fracture patients requiring simultaneous management of osteoporosis and cardiopulmonary function)^2^.

With the surge in demand for home-based rehabilitation (globally, up to one-third of the population, or 2.41 billion people, may benefit from rehabilitation during illness or injury, and 68% of chronic disease rehabilitation patients in the U.S. rely on remote guidance), online health communities have emerged as critical channels for patients to access self-management solutions^3^. These platforms transcend temporal and spatial barriers by integrating patient-provider interaction UGC, such as Q&A data and rehabilitation diaries^4^. However, platform data utilization presents dual contradictions: patient-side inefficiencies due to unstructured Q&A data (e.g., a 37% duplication rate in queries like “post-knee replacement swelling management”) and platform-side resource waste from insufficient fine-grained knowledge mining^5,6^.

Our research aims to systematically construct a dynamic “disease-symptom-rehabilitation measure” association framework, transforming tacit experiential knowledge from unstructured UGC into explicit representations to provide rehabilitation decision support while enabling knowledge sharing. To achieve this, we designed a BERT-BiGRU-attention model to extract relationships among three entity types—disease, symptom descriptions, and rehabilitation measures—from two million rehabilitation Q&A entries. Entity clustering further uncovered additional associative knowledge. This work holds significant value for supplementing and integrating clinical rehabilitation data, overcoming the static limitations of traditional guidelines. Additionally, the extracted knowledge enables dynamic matching of rehabilitation measures with patient profiles, empowering patients in self-managed rehabilitation.

## Related work

### Research on knowledge extraction in online health communities

A massive user base in online health communities generates a wealth of user-generated content containing a plethora of untapped information. Extracting knowledge from this can offer new insights and solutions to health issues. For instance, extracting important relationships between diseases, symptoms, and medications^7,8^, analyzing the distribution of topics within breast cancer communities, tracking topic dynamics, and observing changes in disease severity over time^9^, using user comments to make wiser choices in selecting experts^10^, and identifying depression emotions^11^. Additionally, extraction of medicine knowledge from online health communities plays a crucial role in the field of relationship extraction. Examples include extracting adverse drug events (ADEs) from user-generated content in online health communities^12,13^, identifying new indications for drugs beyond their package inserts^14–16^, detecting prescription drug abuse^17^, extracting relationships between drugs and their effects using the Medical Dictionary^18^, and extracting dietary recommendation knowledge from online health websites using rule-based methods, among others^19^.

In the context of multimodal rehabilitation knowledge mining, the vast unstructured user-generated content (UGC) from online rehabilitation Q&A communities, including colloquial expressions and metaphors, provides novel pathways to explore relationships among diseases, symptoms, and rehabilitation measures. This supports the construction of dynamic rehabilitation knowledge graphs, integrates UGC with clinical guideline knowledge systems, and enhances rehabilitation decision-making.

### Research in medical knowledge discovery

Early scholars primarily used pattern matching and machine learning techniques to extract disease-drug relationships from medical texts. Pattern matching relied on syntactic analysis and expert-defined rules, yielding low recall rates. Methods employing syntax analysis and semantic dependencies have been widely adopted to extract relationships among diseases, symptoms, and drugs from diverse datasets. For instance, Iqbal et al. used rule-based extraction to identify drug-side effect relationships from electronic medical records^20^. In tasks like the I2B2/VA challenge, machine learning was applied to extract relationships among medical concepts in clinical records, including problems, examinations, and treatments^21^. Support vector machines and kernel methods have been effective in extracting relationships between chemicals and diseases from PubMed literature^22^. Various machine learning algorithms have also been employed to extract semantic relationships such as cure, prevention, and side effects from clinical records and discharge summaries^23^, as well as relationships related to patients’ medical problems (diseases, examinations, and treatments) from discharge summaries and medical literature^24^. Machine learning methods have been widely applied in extracting adverse drug events (ADEs)^12,13^, new indications for drug labels^14^, drug-drug interactions^25^. Despite these advancements, machine learning remains constrained by the complexity of feature engineering, and thus its performance requires further improvement.

Artificial intelligence, particularly deep learning, has revolutionized various industries. Unlike traditional machine learning methods that require manual features and domain knowledge, deep learning automates feature extraction through multi-layer neural networks, reducing human effort significantly^26^. This technology has found wide application in health information processing. For instance, it has been used to extract Bacteria Biotope events^27^, and classify relationships in medical contexts such as problems-treatment, problems-detection, and inter-medical problem relationships^28^. Deep learning frameworks have also been effective in tasks like chemical-induced diseases^29^, nodule detection^30^, medical labeling, and scanning^31^. Additionally, it has extensive applications in extracting adverse drug events^32^, drug-drug interactions^33^, and the therapeutic effects of drugs on disease^34–36^. It leverages advanced techniques like BERT word vectors and word embeddings to enhance natural language processing capabilities in health informatics^36^.

Current research primarily focuses on structured data sources like electronic medical records, discharge summaries, and medical literature abstracts for extracting disease-related relationships. However, these datasets are small in scale, limiting the breadth of knowledge gained from relation extraction. Moreover, the effectiveness of these methods on large, colloquial, and unstructured text datasets is not optimal. Although some scholars have explored using deep learning to extract disease-related relationships from large, unstructured datasets, there remains untapped potential in utilizing vast question-answer data from online health communities. This data, generated by millions of users, could provide valuable insights to enhance knowledge bases and support clinical decision-making. Given that deep learning is pivotal in natural language processing and the primary method for addressing disease-related relationship extraction, this study aims to address the challenge of extracting knowledge from unstructured online Q&A rehabilitation communities between doctors and patients using deep learning techniques, and to leverage the acquired knowledge to facilitate rehabilitation-related health management.

### Rehabilitation support systems

Rehabilitation support systems drive the intelligent development of disease recovery through technological innovation and diversified application scenarios. In the realm of personalized rehabilitation recommendations, Al-Remawi and Aburub utilize convolutional neural networks (CNN) to analyze radiomic features (e.g., lymphatic vessel texture) in postoperative breast cancer patients, accurately identifying biomarkers such as VEGF-C^37^. They further generate personalized nutrition plans via intelligent platforms, significantly improving patient rehabilitation outcomes. The integration of remote rehabilitation and IoT technologies expands application scenarios—for example, wearable sensors combined with decision tree (DT) algorithms enable remote ankle rehabilitation systems to synchronize patient motion data through fiber-optic sensors. Based on DT classification models, these systems provide personalized recommendations to optimize training intensity in real-time^1^, thereby reducing reinjury risks.

In the field of sports rehabilitation, researchers employed the KIMORE dataset to evaluate chronic low back pain rehabilitation. Using a GCN-LSTM model to analyze five types of lumbar movements (e.g., trunk rotation, squats), the prediction error for scoring was reduced by 40% compared to traditional methods^38^. In sports injury optimization, wearable knee sensors monitor gait symmetry, while immersive training enhances patient engagement. Combined with LSTM-based reinjury risk prediction^39^, secondary surgery rates are markedly reduced.

For AI-driven psychological rehabilitation interventions, technologies such as natural language processing (NLP), random forests, and long short-term memory (LSTM) networks provide real-time support for cancer patients. For example, Woebot (based on cognitive behavioral therapy) and the meditation app Headspace dynamically adjust CBT content through interactive dialogues and sentiment analysis, integrating NLP and random forest (RF) algorithms to significantly alleviate anxiety and depressive symptoms in cancer patients^40^.

In the domain of knowledge base construction and system integration, researchers emphasize the importance of structured knowledge bases. The intelligent medical rehabilitation system proposed by Hou et al. collects patient physiological data in real time through an IoT sensing layer, integrates cross-institutional information sharing via a cloud platform, and supports remote personalized protocol design by physicians (e.g., exercise intensity control for osteoporosis patients)^2^. Additionally, its rehabilitation knowledge base query module transforms unstructured doctor-patient Q&A (UGC) into standardized clinical guidelines. Similarly, Wang et al. developed a remote stroke rehabilitation system that combines a MySQL database with a PHP development framework to automate rehabilitation prescription generation, video guidance, and training reports, reducing reliance on offline resources and alleviating regional healthcare disparities. This system enabled 68% of chronic disease patients in the U.S. to complete training via remote guidance^41^. These integrated knowledge systems play a critical role in addressing the static limitations of traditional guidelines.

However, the field still faces multiple challenges: low adoption rates of smart rehabilitation devices and inconsistent evaluation metrics for rehabilitation knowledge across clinical scenarios. Future development must focus on the innovative integration of technology and knowledge. Technologically, federated learning and edge computing can enable low-latency, high-privacy remote rehabilitation management. For standardization, interdisciplinary frameworks involving clinicians, data scientists, and ethicists are needed to establish unified evaluation systems. In knowledge sharing, multi-modal rehabilitaton knowledge (e.g., text, imaging data, sensor signals, and patient reports) should be mined and fused through diverse channels to promote collaborative refinement of rehabilitation knowledge systems. Building on this, our study proposes a BERT-BiGRU-attention model to extract implicit rehabilitation knowledge from user-generated content (UGC) in online health Q&A communities. Through clustering analysis, we categorize this knowledge into groups (e.g., disease-symptom clusters, intervention recommendation clusters), revealing latent associations. This work holds significant value for enhancing rehabilitation knowledge systems, empowering patient self-management, supporting personalized clinical decision-making, and improving health education efficiency.

## Methodology

**Fig. 1:**
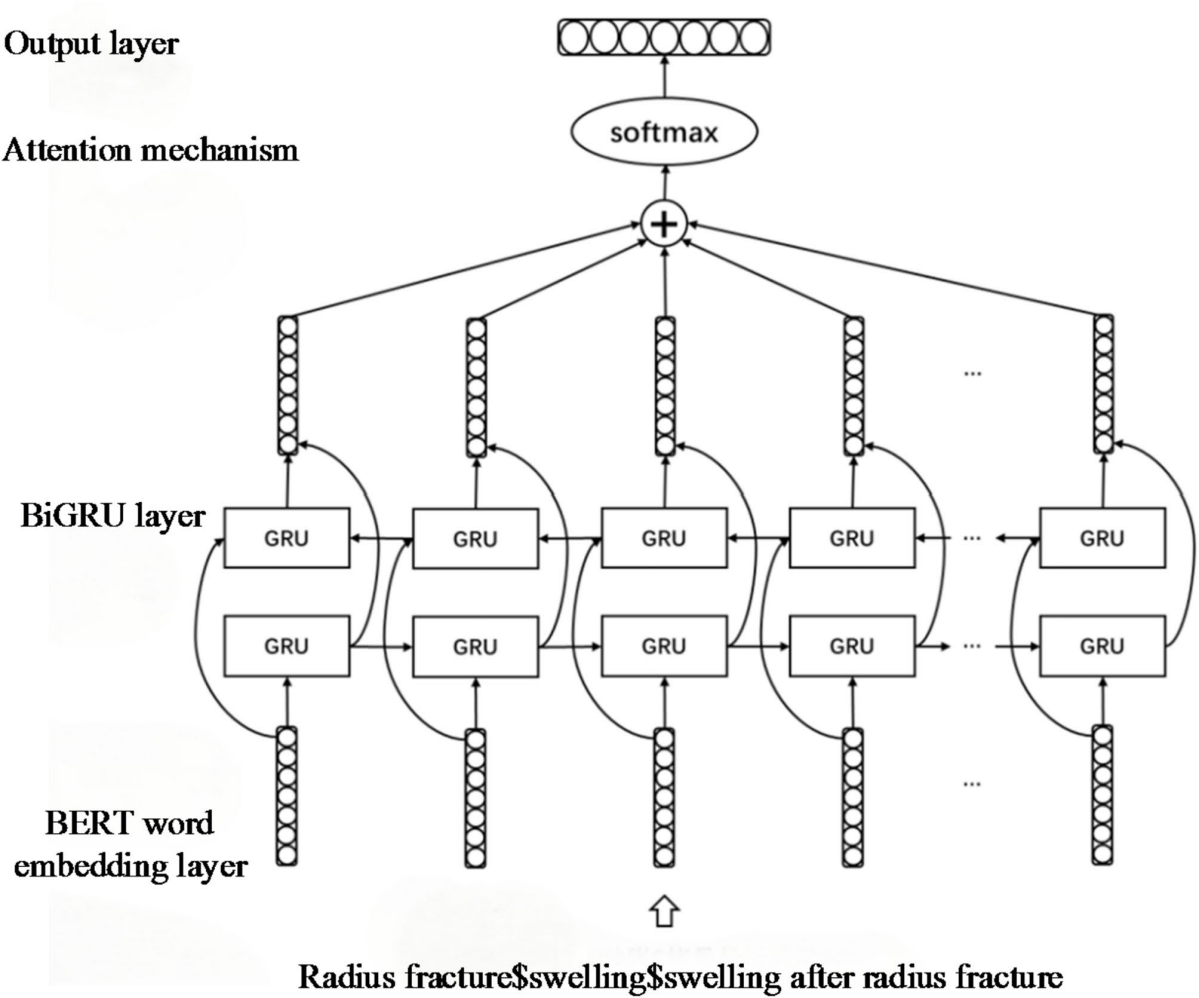
The BERT-BiGRU-attention model for extracting rehabilitation knowledge relationships.

Build a rehabilitation knowledge extraction model using BERT-BiGRU-attention architecture, comprising input, BERT word embedding, BiGRU, attention, and output layers (see Fig. 1).

The BERT-BiGRU-attention model has significant advantages in extracting relationships among diseases, symptoms, and rehabilitation measures. BERT (Bidirectional Encoder Representations from Transformers) learns bidirectional semantic representations from large-scale pre-training on a vocabulary enriched with medical entities and dictionaries. When combined with annotated data, it effectively captures complex contextual associations in medical texts through deep semantic reasoning. For example, implicit relationships between disease terms (e.g., post-stroke hemiplegia) and symptoms (e.g., finger stiffness) can be more accurately encoded via BERT’s contextual embeddings. The Bidirectional Gated Recurrent Unit (BiGRU) captures both forward and backward sequential dependencies while offering higher computational efficiency than BiLSTM^18^, making it suitable for modeling long-distance associations in lengthy medical narratives. In a sentence such as “Long-term finger stiffness after post-stroke hemiplegia requires enhanced hand functional exercises,” BiGRU effectively models the disease-symptom relationship (post-stroke hemiplegia → finger stiffness) and links it to the rehabilitation measure (enhanced hand functional exercises). The attention mechanism dynamically allocates weights to highlight critical semantic segments^27^. For instance, in the above sentence, the model automatically focuses on the connections between post-stroke hemiplegia, finger stiffness, and enhanced hand functional exercises, mitigating interference from irrelevant terms or non-correlated descriptions.

Therefore, our research employs the BERT-BiGRU-attention model, leveraging the tripartite advantages of pre-trained semantic understanding, sequential modeling, and feature prioritization to efficiently address complex relationship extraction among diseases, symptoms, and rehabilitation measures in medical texts.

First, the input layer preprocesses the corpus by concatenating patient questions with doctor responses to create a complete relationship extraction corpus. Next, the corpus containing only one type of entity is filtered out, and annotated using a novel tagging method, marking entity positions with special symbols like " relation@entity1$entity2$text “.

After annotating the corpus, the text is vectorized in the word embedding layer. The rehabilitation Q&A community corpus presents challenges such as non-standard vocabulary, varied grammar, and polysemy. Patients typically describe their conditions and ask questions first, with doctors responding by analyzing and advising based on these descriptions. Doctors often use pronouns to refer to patient conditions. Utilizing the BERT model is essential to capture contextual information through bidirectional Transformer architecture, enhancing the precision and effectiveness of word representations.

Thirdly, after obtaining word vectors, they are fed into the neural network for training. The rehabilitation knowledge corpus features entities in both questions and answers, and only parts of the text indicate their relationship amid considerable noise. Integrating BiGRU networks to manage long-text dependencies and attention mechanisms to prioritize relevant text enhances entity relationship prediction by filtering out irrelevant noise.

Finally, vectors learned from the neural network are processed through a fully connected layer and softmax normalization. The label with the highest probability determines the classification label for the corpus, resulting in a triple (entity1, entity2, relationship). To facilitate knowledge extraction, all triples are categorized and summarized into a relationship dictionary based on entity relationships, capturing rehabilitation knowledge across different relationships. Additionally, to enhance intuitive understanding, a visual relationship network graph is generated as output.

### Input layer in relationship extraction model

The input layer of the rehabilitation knowledge relationship extraction model mainly involves data preprocessing and annotation. In medical Q&A scenarios, there is typically a strong contextual link between patient questions and doctor responses. Extracting relationships between entities solely from questions or answers presents challenges. For instance:

Patient Question: “I have pain below the patella when squatting or going up and down stairs. What could it be?”

Doctor Response: “Based on your description, it could be arthritis. You should rest and take care of it. You can apply traditional Chinese medicine or medicated wine locally, take oral trauma pills, and use anti-inflammatory and analgesic drugs. Combining these measures with traditional Chinese medical therapy can help alleviate and improve the condition.”

Patient questions alone provide a corpus for extracting “symptom” entities without relationships between them. Doctor responses alone provide a corpus where relationships between “disease” and “rehabilitation measures” can be extracted. Combining patient questions and doctor responses into a unified corpus allows extraction of relationships involving three types of entities. Therefore, for comprehensive rehabilitation knowledge mining, patient questions and doctor responses are concatenated. Additionally, cases where the concatenated corpus contains only one type of entity are excluded.

During the relationship extraction phase, the corpus undergoes annotation to identify two types of entities and their relationships. These relationships include DS (symptom of disease), SFD (suitable for disease), NSFD (not suitable for disease), and UKN (unknown). Annotated corpus entries are formatted as “relationship@entity1$entity2$text.”

In relationship extraction tasks, two common annotation methods for corpora are used: (1) Only annotating the two entities of interest; (2) Position-marker annotation: Annotating both entities with special markers indicating their original positions in the corpus. This approach aids in accurately identifying sentences with implicit entity relationships. To enhance model learning effectiveness, it’s crucial to manage entity order in the corpus. Thus, in our model, we employ different symbols for marking entity positions. Specifically, “#” denotes the position of entity 1, and “*” denotes the position of entity 2. For instance, when annotating the relationship between “arthritis” and “trauma pills” in a corpus, the annotation example is as follows: “SFD@arthritis$trauma pills$ Consideration is #. You need to rest and take care of it, you can apply traditional Chinese medicine or medicated wine locally, and take * internally.” (Note: This is a simplified example; actual experimental corpora retain complete patient questions and doctor responses.)

### BERT word embedding layer in relationship extraction model

After obtaining annotated corpora, the textual data is passed into the BERT word embedding layer to get vector representations. In rehabilitation knowledge relationship extraction in Q&A communities, there are some issues: first, the grammar and structure of the corpora are non-standard; second, the same vocabulary can have different meanings and entity types depending on the context, which can affect relationship extraction results. Therefore, using the BERT model’s bidirectional Transformer architecture helps provide more accurate vector representations based on contextual meanings.

In a Q&A setting, patients first describe their condition and ask questions, with doctors responding by analyzing and offering guidance based on the patient’s account. For instance:

Patient: “When I was young, I fell from upstairs and possibly broke a bone in my waist. I applied some random treatment and didn’t go to the hospital. Now, I occasionally feel pain. I’m a 22-year-old male. What’s the best examination if I want to get a check-up?”

Doctor: “In your situation, you could start with an X-ray, but an MRI would be better. It gives a clearer view of your lumbar vertebrae and surrounding tissues. Make sure to sleep on a firm mattress and avoid long periods of standing or sitting.”

Here, the phrase “In your situation” refers to the patient’s specific condition. To accurately represent the semantics of such passages, word embedding methods must take into account contextual information and manage long-text dependencies. BERT, with its bidirectional transformers and multi-head self-attention, is well-equipped for this. Thus, BERT is used as the word embedding layer, utilizing Google’s BERT-base Chinese model with 12 Transformer layers. The sentence length is set to 200, with each character represented by a 768-dimensional feature vector.

### BiGRU + attention layer in relationship extraction model

After obtaining word embedding vectors from the corpus, they are fed into the neural network for model training.

In the task of extracting rehabilitation knowledge relationships from online Q&A communities, the two entities involved are often found in the patient’s question and the doctor’s response. For example:

Patient: “I’m an athlete. After running last week, the outer side of my ankle started hurting, and the ligament above the ankle is painful whenever I move it. What’s the issue? Any treatment advice?”

Doctor: “From your description, it seems to be a sports injury. It’s recommended to get an MRI of the ankle joint…”.

In this example, “pain on the outer side of the ankle” is a symptom, “sports injury” is a disease, and “MRI of the ankle joint” is a rehabilitation measure. These entities appear in both the question and the response. To extract relationships between them, it’s essential to consider the context and handle long-text dependencies. Thus, bidirectional gated recurrent units (BiGRU) are chosen for analysis.

In relation extraction tasks, due to noisy text, only part of the corpus typically expresses the relationship between two entities. Using an attention mechanism helps increase the weight of relevant text, filtering out noise and enhancing the model’s ability to predict relationships. The structure of this layer is shown in Fig. 1.

*Step 1* The BiGRU layer receives the BERT word embeddings as input for each corpus, represented as X ∈ R^(200 × 768), where the sentence length is 200 and each word vector is 768-dimensional. X passes through forward and backward GRU layers, each with 32 hidden units. The output feature vectors from both GRU layers are concatenated to get H ∈ R^(200 × 64).

*Step 2* Introducing the attention mechanism:

1 $${\text{M }}={\text{ tanh}}\left( {\text{H}} \right)$$

2 $$\alpha \,=\,{\text{softmax}}({\omega ^{\text{T}}}{\text{M}})$$

3 $${\text{c }}={\text{ H}}{\alpha ^{\text{T}}}$$

Where ω is the training parameter, and c∈R^64^.

### Output layer in the rehabilitation knowledge relationship extraction model

Relationship extraction in deep learning is essentially a classification task. The vectors learned from the neural network are passed into a fully connected layer to reduce the output dimensions. The vector’s dimensionality corresponds to the number of classification labels. Then, softmax is applied to normalize the vectors, ensuring each output element is within the range [0, 1].

4 $$\:\text{f}\left(\text{z}\text{j}\right)\:=\frac{{e}^{{z}_{j}}}{{\sum\:}_{i=1}^{n}{e}^{{z}_{i}}}$$

Where z_j_ is the j-th element of the vector from the fully connected layer, n is the number of classification labels, and f($\:{\text{z}}_{\text{j}}$) represents the probability of classifying the corpus as the j-th label. The label with the highest probability is selected as the classification label for the corpus.

After extracting relationships from each corpus, triples (entity1, entity2, relationship) are generated. These triples are organized into DS, SFD, and NSFD dictionaries, indexed by disease name. The DS dictionary includes diseases and their symptoms, the SFD lists diseases with suitable rehabilitation measures, and the NSFD lists unsuitable measures. Additionally, relationship network diagrams are provided for better visualization.

### Rehabilitation knowledge clustering analysis model

**Fig. 2:**
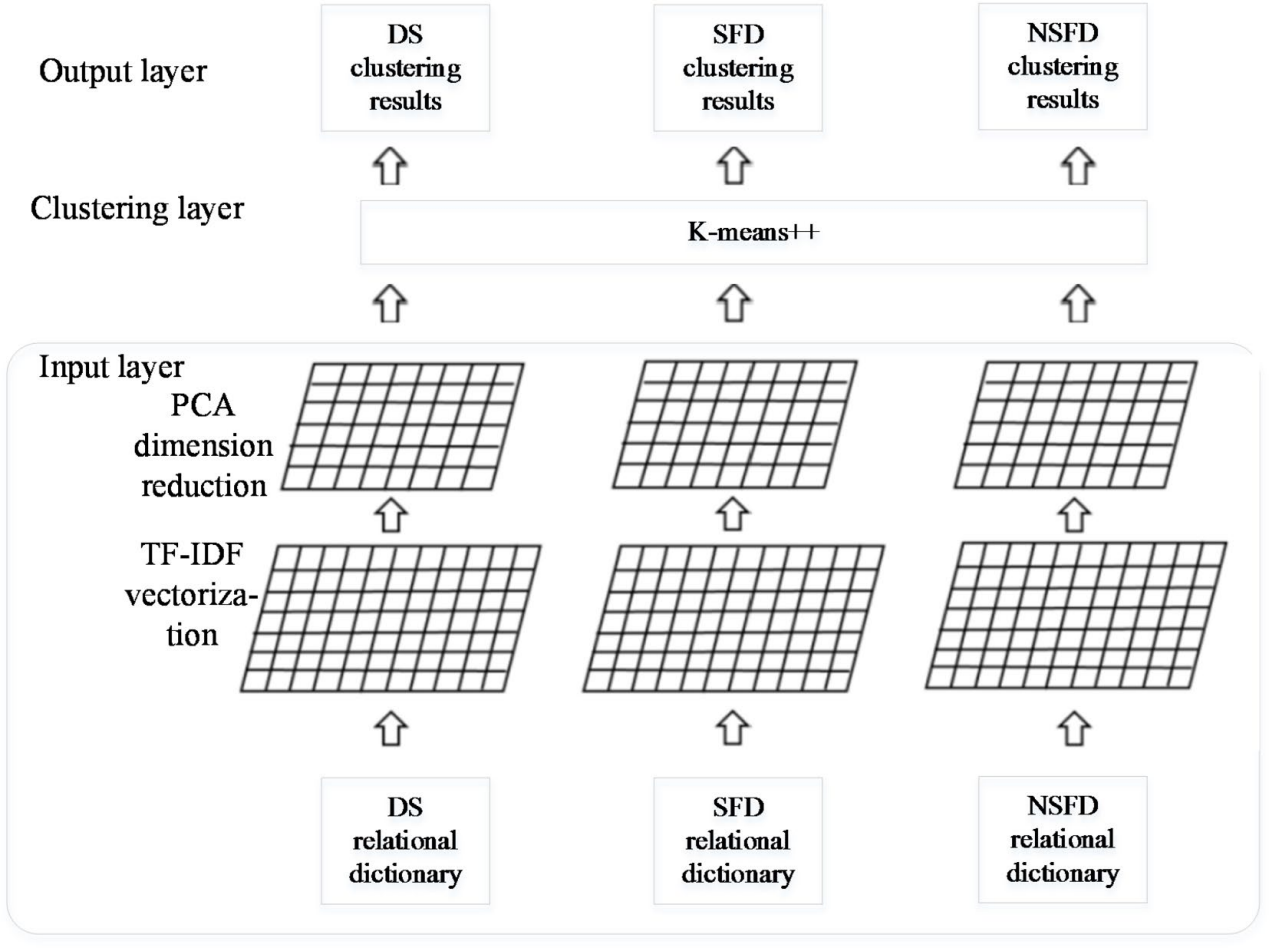
Rehabilitation Knowledge Clustering Analysis Model in Q&A Communitie.

In the rehabilitation Q&A knowledge mining task, clustering analysis is required after knowledge extraction. A clustering analysis model based on kmeans + + is constructed to cluster diseases in the DS, SFD, and NSFD dictionaries obtained from the relationship extraction phase. This model includes an input layer, clustering layer, and output layer, as shown in Fig. 2.

The following sections will explain each layer in the Rehabilitation Knowledge Clustering Analysis Model.

#### Input layer in the rehabilitation knowledge clustering analysis model

In the rehabilitation knowledge relationship extraction model, three relationship dictionaries are obtained and categorized based on entity relationships: DS dictionary, SFD dictionary, and NSFD dictionary. Each dictionary uses the disease name as the key and includes symptoms, suitable rehabilitation measures, and unsuitable rehabilitation measures as values.

In the input layer of the rehabilitation knowledge clustering analysis model, the values of the relationship dictionaries are vectorized using the TF-IDF (term frequency–inverse document frequency) method. This approach helps the clustering algorithm focus on more representative rehabilitation knowledge. For instance, in our corpus, common phrases in the SFD dictionary include “surgical treatment,” “medication treatment,” and “rehabilitation treatment,” where the term “treatment” appears frequently. Using this as a clustering basis may lack representativeness for different diseases, so its weight can be reduced.

Next, principal component analysis (PCA) is used to reduce the dimensionality of the text vectors. Dimensionality reduction helps eliminate noise and unimportant features, enhancing clustering effectiveness.

#### Clustering layer in the rehabilitation knowledge clustering analysis model

After obtaining the dimensionality-reduced text vectors, the kmeans + + algorithm is used for clustering. The traditional kmeans algorithm randomly selects k sample points as initial cluster centers, which can significantly affect the final results. The k-means + + algorithm improves this process by prioritizing points that are farther away from already selected center points when choosing the (*n* + 1)th center point.

#### Output layer in the rehabilitation knowledge clustering analysis model

In the output layer, clustering results from the three relationship dictionaries are output separately.

### Rehabilitation knowledge mining evaluation metrics

Supervised learning algorithms are used for relationship extraction in the rehabilitation Q&A community. In these models, precision, recall, and F-score are commonly used to evaluate performance in relationship extraction in the medical field. Precision (P), recall (R), and F1-score are calculated as follows:

5 $$\:\text{P}=\:\frac{TP}{TP+FP}$$

6 $$\:\text{R}=\:\frac{TP}{TP+FN}$$

7 $$\:\text{F}1-\text{s}\text{c}\text{o}\text{r}\text{e}=\:\frac{2*precision*recall}{precision+recall}$$

In these formulas, TP refers to True Positives, FN to False Negatives, FP to False Positives, and TN to True Negatives. The metrics P and R represent the model’s precision and recall, while the F1-score, which combines P and R, serves as a third evaluation metric.

AUC (ROC Area) evaluates how well a model distinguishes positive and negative classes by plotting True Positive Rate (TPR) vs. False Positive Rate (FPR) across thresholds. A higher AUC (closer to 1) means better performance, suited for balanced data.

AUPR (PR Area) assesses positive class prediction quality by balancing Precision (accuracy) and Recall (coverage) in the PR curve. It excels in imbalanced data (e.g., rare events). AUC highlights overall class separation, while AUPR focuses on precision-recall trade-offs for positives, making them complementary.

All three metrics range from 0 to 1, with higher values indicating better performance.

For clustering analysis in the rehabilitation Q&A community, unsupervised learning algorithms are employed. The silhouette coefficient, denoted as s, evaluates these algorithms by measuring cohesion and separation, even without true cluster labels.

8 $$\:\text{R}\text{e}\text{c}\text{a}\text{l}\text{l}=\:\frac{b-a}{\text{m}\text{a}\text{x}（a，b）}$$

Here, ‘a’ denotes the average distance between a sample point and others in the same cluster, reflecting cohesion, while ‘b’ indicates the average distance to the nearest cluster, reflecting separation. The silhouette coefficient ranges from − 1 to 1, with values closer to 1 signifying better model performance.

## Experimental design for knowledge mining in rehabilitation Q&A community

Two experiments focus on the knowledge mining goals of the rehabilitation Q&A community: relation extraction and clustering analysis. In the relation extraction experiment, a preprocessed Q&A corpus is annotated with three types of entity relations: symptoms linked to diseases, suitable rehabilitation measures, and unsuitable ones. The BERT-BiGRU-attention model is used for relation extraction. The annotated dataset is split into training and testing sets, with optimal hyperparameters chosen for training. The model’s performance is evaluated on the test set and compared to other baseline models to validate its effectiveness.

Using the results from relation extraction, the identified relations are organized into a relation dictionary, which is then used for clustering analysis. This analysis clusters diseases based on their symptoms and appropriate or inappropriate rehabilitation measures. A specific disease category is analyzed to offer recommendations for managing rehabilitation knowledge in online health communities.

### Experimental dataset

The experimental dataset is obtained from the 120ask website (https://www.120ask.com), one of China’s largest online medical consultation platforms, featuring extensive physician resources and a well-developed management system. It has a specific Q&A section for “Rehabilitation Medicine,” where orthopedic postoperative rehabilitation questions are especially detailed and useful for knowledge mining.

For this experiment, data from March 2017 to October 2022 was collected through web scraping in the “Orthopedic Postoperative Rehabilitation” section, resulting in 37,879 entries compiled into a CSV file. The dataset includes patient information, question timestamps, titles and content, number of responding doctors, their account and professional details, response timestamps, and answer content.

In this experiment, the “question title” and “question content” refer to patient inquiries on a Q&A website, while the “answer content” includes doctors’ responses. Each patient inquiry usually has multiple doctor responses, which are fully collected during data scraping. Together, a patient inquiry and its corresponding responses form one data record.

### Data preprocessing

The steps for preprocessing data to explore rehabilitation knowledge are as follows: First, filter the corpus to remove irrelevant content, such as questions about nucleic acid testing for fractures or the cost of fixed supports. Next, correct typographical errors and special characters in the medical Q&A corpus, which may contain mistakes from speech-to-text technology. For relation extraction experiments, identified medical entities from named entity recognition are used as input. This involves combining the “question title,” “question content,” and “answer content” into a single line. Finally, filter out instances with only one type of entity to create the final corpus for relation extraction.

### Rehabilitation knowledge mining experiment process

#### Experimental environment

The experiment uses Python 3 and TensorFlow 1.15.0, a Google open-source platform for machine learning and deep learning, applicable in fields like computer vision and natural language processing.

#### Entity definition and experimental procedure

For rehabilitation knowledge mining, three entities are defined: diseases, symptoms, and rehabilitation measures. Simple entities like “going up and down stairs” were labeled as rehabilitation measures. Relationships between rehabilitation measures and diseases are categorized as “inappropriate” (e.g., “reduce,” “avoid”) or “appropriate” (e.g., “increase,” “improve”). Undefined relationships, noted as UKN, represent unclear links, such as between “fractured ankle” and treatment options in the question about conservative treatment versus surgery. The definitions of entities and relationships are shown in Tables 1 and 2.

**Table 1:** Named entity definition in rehabilitation knowledge.

Identifier	Entity Type	Entity Definition	Example
DIS	Disease	Unhealthy abnormal life processes	Radial fracture, ankle soft tissue injury
SYM	Symptom	Subjective abnormal sensations or objective pathological changes caused by diseases	Swollen ankles, restricted knee joint movement
TRE	Treatment	Professional diagnostic and treatment methods adopted for the discovery and treatment of diseases, as well as daily activities that are beneficial or detrimental to disease recovery	Patellar tendon repair surgery, elevating the affected limb, massaging the affected area

**Table 2:** Relationship definition in rehabilitation knowledge.

Identifier	Relationship Definition	Entity 1	Entity 2
DS	symptom of disease	DIS	SYM
SFD	suitable for disease	DIS	TRE
NSF	not suitable for disease	DIS	TRE
UKN	unknown	-	-

**Table 3:** Annotation statistics of rehabilitation knowledge relationship extraction Corpus.

Relationship Category	Number of Annotations
DS	901
SFD	3602
NSFD	497
UKN	235

Before extracting rehabilitation knowledge relationships, the annotated corpus must include four relationship types from Table 2. The format is: Relationship@Entity1$Entity2$Text. To clarify entity positions, “#” marks entity 1 and “*” marks entity 2. For example, in “considering arthritis, apply traditional Chinese medicine or medicated wine externally, and take bruise pills internally,” the annotation for “arthritis” and “bruise pills” would be: “SFD@arthritis$bruise pills$Considering is #, apply traditional Chinese medicine or medicated wine externally, and take * internally.” in total, 5,235 pieces of corpus were annotated, with various relationship types detailed in Table 3.

**Table 4:** Size of training, validation, and test sets.

Category	#DS	#SFD	#NSFD	#UKN	Total
Training Set	541	2161	298	141	3141
Validation Set	180	720	100	47	1047
Test Set	180	721	99	47	1047
Total	901	3602	497	235	5235

As shown in Table 3, the categories in this experimental dataset are imbalanced. To prevent bias from uneven partitioning, the dataset is shuffled and divided into training, validation, and test sets using stratified sampling in a 6:2:2 ratio, ensuring consistent category distribution. The training set is for model training, the validation set is for tuning hyperparameters, and the test set is used to evaluate the model’s performance against a baseline. Table 4 details the sizes and counts of each relationship category in these sets.

In each experiment round, precision, recall, and F1-score are calculated for each relationship type, as well as overall metrics for the four relationship types, to evaluate classification effectiveness. Hyperparameter tuning occurs on the validation set, using these metrics to select optimal parameters. After determining the best hyperparameters, the model is assessed using the test set. Additionally, four control experiments are conducted with models like BERT-BiGRU, BiGRU-attention, CNN, and SVM for rehabilitation knowledge extraction, comparing their performance based on test set metrics to highlight the experimental model’s effectiveness.

#### Hyperparameter tuning and setting

**Fig. 3:**
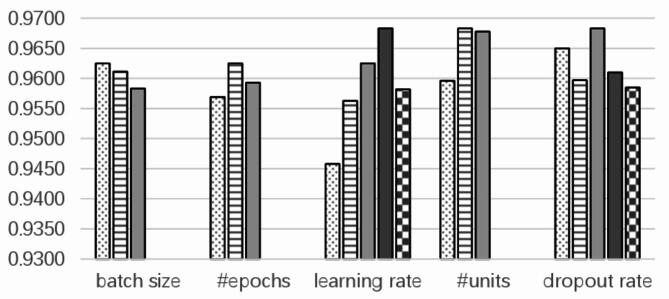
Influence of Various Hyperparameters on F1-score.

To improve model performance, the training set is used first for training, followed by hyperparameter tuning on the validation set. The F1-scores for relationship extraction, based on different hyperparameter values from the validation set, are visually represented in Fig. 3.

The parameter configurations in the Fig. 3 include batch sizes of 16, 32, and 64, training epochs spanning 30, 40, and 50 iterations, learning rates set to 0.0, 0.005, 0.001, 0.0005, and 0.0001, hidden layer units configured with 16, 32, or 64 neurons, and dropout rates tested across five levels (0, 0.1, 0.2, 0.3, 0.4) for hyperparameters evaluation. Figure 3 shows that for the BERT-BiGRU-attention relation extraction model, the batch size is inversely related to the F1-score when using Mini Batch gradient descent. For the other four hyperparameters, excessively high or low values negatively impact model training. The optimal hyperparameters for relation extraction on the validation set are a batch size of 16, epochs of 40, a learning rate of 0.0005, 32 hidden neurons, and a dropout rate of 0.2.

#### Rehabilitation knowledge clustering analysis experimental design

After extracting rehabilitation knowledge relationships, further clustering can be done. This analysis uses three relationship dictionaries (DS, SFD, and NSFD) from the extraction experiment, employing symptoms linked to diseases and appropriate or inappropriate rehabilitation measures for clustering.

To ensure effective clustering, the three relationship dictionaries from the extraction experiment are verified, and records with errors are removed to prepare the input for cluster analysis, the Table 5 illustrates the DS Dictionary as an example. Each dictionary’s values are clustered using the Kmeans + + algorithm, with the silhouette coefficient helping to determine the optimal number of clusters by adjusting hyperparameters. For each hyperparameter, the model performs five iterations, averaging the silhouette coefficients for evaluation. For the DS dictionary, the clustering category numbers are incremented by 1, revealing that more categories lead to higher evaluation metrics. However, to ensure adequate disease representation, the final category number is set at 15. For the SFD dictionary, 20 categories are chosen for optimal clustering, while the NSFD dictionary is clustered into 6 categories for the best results. Ultimately, diseases in all three dictionaries are clustered based on these optimal numbers, and the results are outputted.

**Table 5:** Cluster analysis input dictionary.

Key	Type	Size	Value
Galeazzi Fracture	list	3	[‘Displacement’, ‘Separation’, ‘Angular deformity’]
Joint Capsule Contraction	list	3	[‘Inability to rotate palm’, ‘Incomplete wrist flexion and extension’, ‘Decreased joint mobility’]
Arthritis	list	3	[‘Pain’, ‘Bone cracking’, ‘Knee sliding’]
Medial Malleolus Fracture	list	3	[‘Pain’, ‘Swelling’, ‘Sensation of pain’]
Right Forearm Radius Deformity	list	3	[‘Slight enlargement’, ‘Slight bone deformation’, ‘Local swelling’]
Right Fibular Distal Fracture	list	3	[‘Pain’, ‘Slight ankle deformation’, ‘Joint stiffness’]
Right Leg Ligament Tear:	list	3	[‘Pain’, ‘Aching’, ‘Joint stiffness’]
Tri-malleolar Fracture	list	2	[‘Inability to squat’, ‘Adhesions’]
Incomplete Paraplegia	list	2	[‘Limited lower limb mobility’, ‘Slightly soft tendons’]
Anterior Cruciate Ligament Avulsion Fracture	list	2	[‘Pain’, ‘Slight depression of tibial plateau’]
Strain	list	2	[‘Pain’, ‘Swelling’]

The number of diseases included in each dictionary is as follows Table 6.

**Table 6:** Statistics of rehabilitation knowledge clustering analysis corpus.

Dictionary Category	Number of Included Diseases
DS	266
SFD	393
NSFD	173

## Experiment results and analysis

### Experiment results and analysis of relationship extraction

The Table 7 displays the results of the relationship extraction experiment using the BERT-BiGRU-attention model with optimal hyperparameters.

**Table 7:** Example of relationship extraction experiment output.

Entity 1: Lumbar muscle strain
Entity 2: Fatigue
Health consultation description: For example, when I bend over and then straighten up, I clearly feel a *, not pain, and I notice that changing the posture of my waist also produces this sensation. What’s going on? Doctor’s response: In cases like yours, it could be #, or it could be a herniated disc, causing symptoms by compressing the nerve. It is recommended that you go to the hospital for an X-ray or CT scan to confirm whether you have symptoms of a herniated disc.
The predicted entity relation is: DS
Entity 1: Fracture of the right radial head
Entity 2: Calcium supplementation
Health consultation description: #. Some doctors say surgery is not needed, while others say it is. Would like to hear opinions. Thank you. Doctor’s response: Condition analysis: Hello, for this type of fracture, it should first be reduced, then stabilized, and then undergo rehabilitation treatment. Guidance: Restore the original function. It is recommended that you pay attention to * and supplement your diet during recovery, which can better promote bone growth.
The predicted entity relation is: SFD
Entity 1: Fracture
Entity 2: Arm movement
Fractured radius. How long until I can move it? Is it serious? Guidance: If it’s #, it usually takes a relatively long time to recover, typically at least about three months. If you have already had it reduced and immobilized, you just need to minimize * to prevent a secondary fracture.
The predicted entity relation is: NSFD
Entity 1: Femoral shaft fracture
Entity 2: Removal surgery of steel nail
It has been # for a year. Can I * now? I can walk normally now, without any pain. Doctor’s response: Normally, a fracture should have healed well after a year, and the internal fixation can usually be removed. I suggest you go to the hospital for another check-up and see if the fracture has completely healed.
The predicted entity relation is: UKN

From Table 7, it is evident that the two entity types for relation extraction are found in patient questions and Doctor responses. the BERT-BiGRU-attention model effectively identifies implicit entity relations within longer sentences. For example, it correctly recognizes the relationship between “reduce” “arm movement” and “fracture” as inappropriate (NSFD). For rehabilitation measures, the model accurately assesses their suitability (SFD) with diseases, such as “right radial head fracture” and “calcium supplementation.” additionally, when Doctors speculate on possible diseases based on patient symptom descriptions, the model can identify the DS relationship, as seen with “lumbar muscle strain” and “fatigue.” the final evaluation results of the relation extraction model are presented in Table 8.

**Table 8:** Result of rehabilitation relationship extraction based on BERT-BiGRU-attention.

Relationship categories	precision	recall	F1-score	AUPR	AUC
DS	0.9454	0.9611	0.9532	0.9409	0.9581
SFD	0.9819	0.9792	0.9805	0.9211	0.9810
NSFD	0.9238	0.9700	0.9463	0.9119	0.9473
UKN	0.9024	0.7872	0.8409	0.8347	0.8654
Total	0.9665	0.9666	0.9663	0.9315	0.9667

The BERT-BiGRU-attention model achieved an overall F1-score of 96.63%, and AUPR of 93.15%, AUC of 95.81% on the test set. Excluding unknown class relationships (UKN), it scored over 90% for each relationship category. However, the recall for unknown relationships was low, likely due to a limited dataset that hindered the model’s learning. the SFD relationship category represented 68.8% of the corpus, where the model performed best. After extracting entity relationship triplets, they were categorized into DS, SFD, and NSFD relationships. This resulted in a DS relationship dictionary containing 266 diseases and their corresponding symptoms, as shown in Table 9.

**Table 9:** DS relationship dictionary (Partial).

DS_dic -Dictionary (266 elements)			
Key	Type	Size	Value
Clayton’s fracture	list	3	[‘Displacement’,‘Separation’,‘Angular deformation’]
Joint capsule contracture	list	3	[‘Inability to flip the palm’,‘Inadequate wrist flexion and extension’,‘Decreased joint mobility’]
Arthritis	list	3	[‘Pain’,‘Joint cracking’,‘Sliding in the knee’]
Fracture of the medial malleolus	list	3	[‘Pain’,‘Swelling’,‘Sensation of pain’]
Deformity of the right ulnar bone	list	3	[‘Slight enlargement’,‘Slight change in bone shape’,‘Local swelling’]
Fracture of the distal end of the right fibula	list	3	[‘Pain’,‘Swelling of the ankle’,‘Turned purple’]
Torn posterior ligament in the right leg	list	3	[‘Pain’,‘Soreness’,‘Joint stiffness’]
Triquetral fracture	list	2	[‘Unable to squat’,‘Adhesion’]
Incomplete truncation of the hymen	list	2	[‘Limited lower limb mobility’,‘Tendons are a bit soft’]
Avulsion fracture of the anterior cruciate ligament	list	2	[‘Pain’,‘Slight depression of the tibial plateau’]
Strain	list	2	[‘Pain’,‘Swelling’]

The SFD (Suitable for Disease) relationship dictionary includes 401 diseases and their corresponding rehabilitation measures, as shown in Table 10.

**Table 10:** SFD relationship dictionary (Partial).

SFD_dic -Dictionary (401 elements)			
Key	Type	Size	Value
Fracture of the right ankle bone	list	3	[‘Muscle contraction training of the calf muscles’,‘Casting’,‘Toe movement’]
Fracture of the distal end of the right fibula	list	1	[‘Surgery’]
Fracture of the right scaphoid bone	list	4	[‘X-ray imaging’,‘Surgery’,‘CT scan’,‘Fixation’]
Torn posterior ligament in the right leg	list	8	[‘Combination of rest and activity’,‘Nutritional support’,‘Joint loosening surgery’,‘Traditional Chinese medicine for promoting blood circulation and relieving pain’,‘Local heat application’,‘Resting’,‘Local warmth’,‘Functional rehabilitation exercises’]
Fracture of the shaft of the right leg	list	4	[‘Functional rehabilitation exercises’,‘Local heat application’,‘Massage’,‘Surgical treatment’]
Right knee sprain	list	3	[‘Medication’,‘Casting’,‘MRI scan’]
Right knee cap sprain	list	1	[‘X-ray imaging’]
Fracture of the right clavicle	list	8	[‘Calcium supplementation treatment’,‘Oral calcium solution’,‘Vitamin D drops’,‘Bone broth’,‘Nutrition’,‘Sun exposure’,‘Carp soup’,‘Calcium gluconate’]
Avulsion fracture of the right patella	list	7	[‘Physical therapy’,‘Functional assessment’,‘Above-the-knee splinting’,‘Physical therapy’,‘Manual therapy for joint loosening’,‘Rehabilitation exercises’,‘Rehabilitation training’]
Peripheral nerve injury	list	2	[‘Nutritional support for nerves’,‘Warm water compress’]
Fracture of the head of the femur	list	3	[‘Functional locking exercise’,‘Immobilization’,‘Bone fixation’]

The NSFD (Not suitable for Disease) relationship dictionary includes 173 diseases and their corresponding inappropriate rehabilitation measures, as listed in Table 11.

**Table 11:** NSFD relationship dictionary (Partial).

Key	Type	Size	Value
Left hand radial fracture	list	1	[‘Intense exercise ‘]
Left hand scaphoid fracture	list	3	[‘ Prolonged sitting or standing’,’ Excessive stress and strain ‘,’ Exposure to cold and damp weather ‘]
Linear fracture of the left radial head	list	2	[‘Weight-bearing training’,’Overexertion ‘]
Fracture of the left radial head	list	4	[‘ Excessive activity’,’ Surgery “,’ Violent sports exercises ‘,’ General activity ‘]

**Fig. 4:**
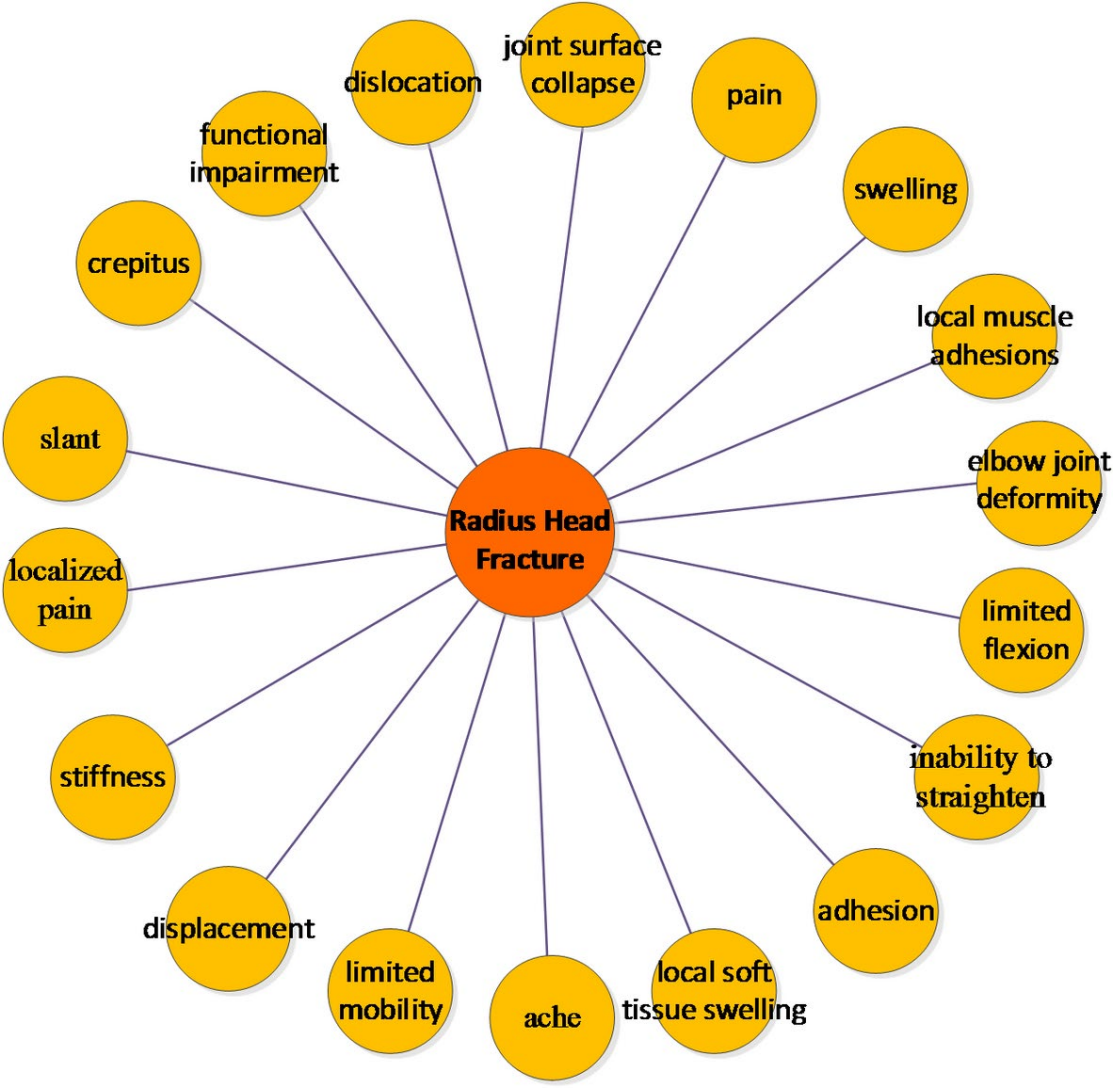
Symptoms of Radial Head Fracture.

To clarify the results of rehabilitation knowledge extraction, we create visual relationship network graphs. Using “radial head fracture” as an example, Fig. 4 demonstrates the associated symptoms, including common manifestations such as swelling and pain, as well as more specific indicators like “elbow joint deformity” and “inability to straighten.”

The Figure 4 shows that common symptoms of “radial head fracture” include swelling and pain. More specific symptoms include “elbow joint deformity” and “inability to straighten.”

**Fig. 5:**
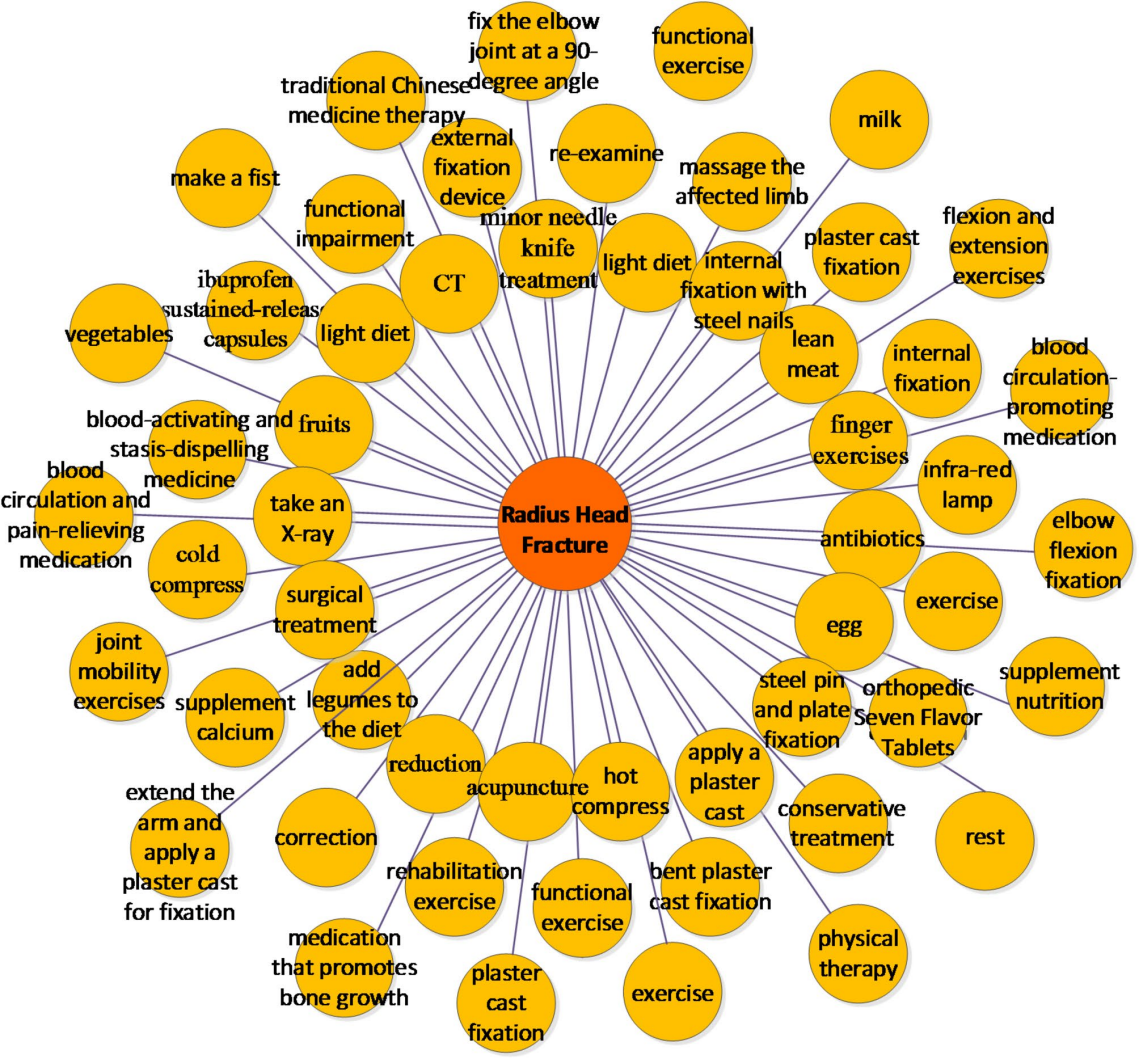
Rehabilitation Measures for Suitable Radial Head Fracture Patients.

**Fig. 6:**
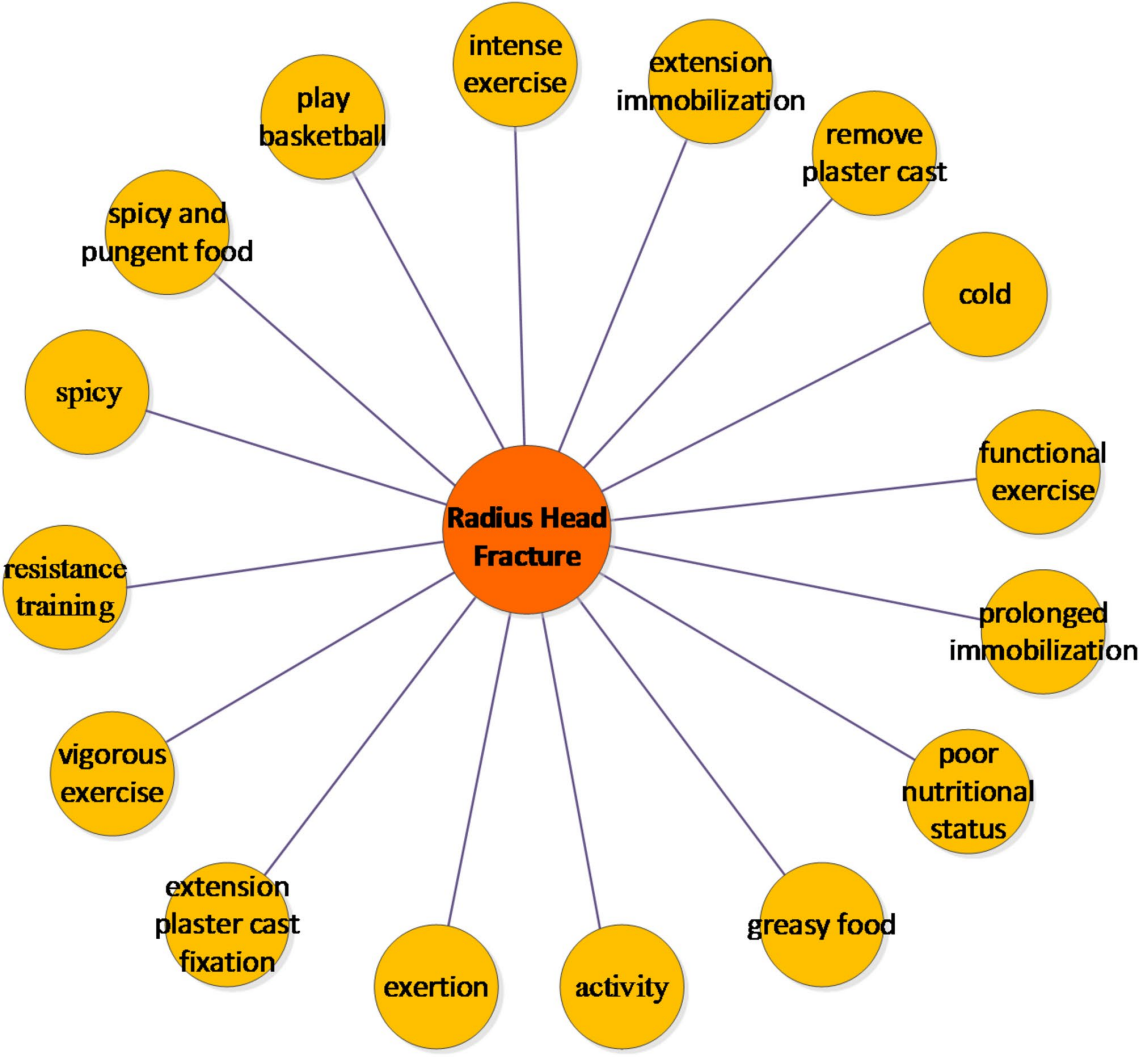
Rehabilitation Measures for Patients with Inappropriate Radial Head Fracture.

Rehabilitation measures suitable and unsuitable for patients with radial head fractures are shown in Figs. 5 and 6. The results in Fig. 5 shows that the model has thoroughly examined rehabilitation measures for radial head fracture patients. Professional treatments include “minor needle knife treatment,” “apply a plaster cast,” and “internal fixation with steel nails.” Patients can also perform simple daily coping measures like “hot compress,” “supplement calcium,” “finger exercises,” and “Massage the affected limb.” For their diet, it’s recommended to include more “vegetables,” “fruits,” “lean meat,” and “milk.”

The results in Fig. 6 indicate that to aid recovery from “radial head fractures,” patients should avoid “greasy food” and “spicy and pungent food,” as well as refrain from playing “basketball” and engaging in “intense exercise.” Contradictions can arise in recovery measures, like “straight plaster cast fixation” and “functional exercises,” due to differing doctor recommendations based on personal experiences. Therefore, online Q&A community managers should focus on extracting rehabilitation knowledge and identifying conflicts, helping doctors provide more cautious advice, which is vital for patient recovery.

### Comparison experiment and analysis of relationship extraction

To assess the feasibility and effectiveness of the model, four comparative experiments were conducted using the same dataset. These included the BERT-BiGRU model without attention, the BiGRU-attention model with traditional word embeddings, the CNN model using conventional neural networks, and the SVM model based on machine learning. The performance of these models was compared with the BERT-BiGRU-attention model developed in this study for extracting relationships in rehabilitation knowledge. The results are presented in Table 12.

**Table 12:** Results of rehabilitation knowledge relationship extraction based on different models.

Relationship categories	precision	recall	F1-score	AUPR	AUC
SVM	0.8454	0.8611	0.8532	0.8316	0.8583
CNN	0.8819	0.8792	0.8805	0.8365	0.8811
BiGRU-attention	0.9238	0.9700	0.9463	0.9102	0.9489
BERT-BiGRU	0.9024	0.7872	0.8409	0.8847	0.8963
BERT-BiGRU-attention	0.9665	0.9666	0.9663	0.9315	0.9667

The model in this study achieved the highest precision, recall, F1-score, AUPR, and AUC as shown in Table 13. the SVM model performed the worst, while the CNN model showed some improvement. In contrast, the recurrent neural network with an attention mechanism excelled, highlighting its strengths in natural Language processing. Our model improved precision, recall, F1-score, AUPR, and AUC by 6.41%, 17.94%, 12.54%, 4.68% and 7.04% compared to the BERT-BiGRU model without attention. This suggests that the attention mechanism helps enhance predictive performance by weighting relevant text and reducing noise. For a detailed comparison of F1-scores across different relationship categories, see Table 13.

**Table 13:** Results of different models on different relationship categories.

Model	UKN-F1	UKN- AUC	DS-F1	DS- AUC	SFD-F1	SFD- AUC	NSFD-F1	NSFD-AUC
BiGRU-attention	0.5714	0.6171	0.8796	0.8914	0.9513	0.9546	0.7835	0.8321
BERT-BiGRU	0.8148	0.8256	0.9415	0.9478	0.9679	0.9683	0.8677	0.8919
BERT-BiGRU-a-ttention	0.8409	0.8654	0.9532	0.9581	0.9805	0.9810	0.9463	0.9473

The Table 13 shows that the BERT-BiGRU-attention model outperformed all other models in extracting relationships. SFD-type relationships were extracted most effectively, while UKN-type relationships were the least effective. Notably, using BERT word embeddings significantly improved the extraction of UKN-type relationships by 26.95% in F1 and 24.83% in AUC compared to the BiGRU-attention model. NSFD-type relationships also saw 16.28% and 11.52% improvement in F1 and AUC respectively, likely due to BERT’s ability to incorporate contextual semantics, which helps classify UKN-type relationships that often appear in questions and better understand terms like “reduce” and “avoid.” Additionally, the inclusion of the attention mechanism notably increased the F1-score for NSFD-type relationships by 7.86%, and AUC for NSFD-type relationships by 5.54%.

### Clustering analysis experimental results and analysis

Using the dictionaries of DS, SFD, and NSFD obtained from the relationship extraction experiment as inputs, clustering analysis experiments were conducted to obtain disease clusters based on symptoms, suitable rehabilitation measures, and unsuitable rehabilitation measures.

1. Results and Analysis of Disease Clustering Based on Symptoms.

Using the DS relationship dictionary, diseases were grouped into 15 categories according to symptom similarity, as shown in Table 14.

**Table 14:** Disease clustering results based on symptoms (Partial).

Category	types of diseases	name of the disease	common symptoms
2	4	Fracture of the middle finger, Fracture of the right radius and ulna, Fracture of the fifth metacarpal bone, Rheumatoid arthritis	limited fist clenching
3	4	Capsular contracture, Distal radius fracture of the wrist, Tendon contracture, Soft tissue contracture	inability to pronate the palm, incomplete wrist flexion and extension
5	13	Arthritis, Meniscus injury, Acute injury, Synovitis, Inflammation, Muscle strain, Knee joint injury, Ligament injury of the knee, Degenerative arthritis, Ligament injury, Osteoarthritis, Chronic injury of the patellar ligament, Patellar dislocation	discomfort in the knee (sliding, pain, soreness, or swelling)
12	4	Synovial lesion or Synovial pathology, Neuropathy or Nerve pathology, Tendinopathy or Tendon pathology, Tenosynovitis	finger pain and pain in the radius bone
13	5	Nerve injury, Tendon strain or Tendon overuse injury, Dislocation, Wrist dislocation, Wrist fasciitis	restricted function of the left forearm
14	5	Internal meniscus injury of the knee joint, Patellar dislocation, Patellar injury, Osteochondritis dissecans of the patella, Patellofemoral osteoarthritis	audible sound and patellar wear
15	190	Triquetral bone fracture, Colles’ fracture, Cervical spondylosis, Cervical vertebra fracture, Rheumatoid arthritis, Pelvic bone fracture, Femoral neck fracture, Fracture of the intercondylar eminence	----------------- ---------------

Clustering diseases by their symptoms from Table 15 results in 14 categories encompassing 76 diseases with distinct symptom features. The 15th category includes 190 diseases, many of which lack typical symptoms, leading to low discriminative power when clustered. Some are less common in online Q&A rehabilitation sections, resulting in few similar symptoms in the data. Notable symptom differences exist among categories, particularly the 3rd, 5th, 12th, 13th, and 14th, where common symptoms can be linked to their locations, aligning with the study’s knowledge mining objectives. For instance, Table 15 presents the diseases in the 2nd category along with their symptom lists.

**Table 15:** List of class 2 diseases and their symptoms.

Index	Type	Size	Value
0	str	1	Fractured middle finger, pain, unable to make a fist
1	str	1	Fractured right radius and ulna, pain, unable to make a fist
2	str	1	Fractured fifth metacarpal bone, dislocated, swollen, unable to make a fist, unable to bend, limited range of motion, stiffness
3	str	1	Rheumatoid arthritis, stiffness in all fingers, pain when making a fist

The second category of diseases features symptoms like “inability to make a fist” or “pain when making a fist,” which suggest limited movement. Conditions such as fractures and arthritis are linked to these symptoms. This clustering analysis aids platform management by allowing it to recommend specialized doctors based on patient-reported symptoms. Moreover, an alert system can notify health community doctors of relevant symptoms in patient questions, guiding them to related diseases during diagnosis. This approach enhances diagnostic accuracy and reduces the risk of misdiagnosis.

(2) Clustering Results and Analysis for Rehabilitation Measures.

Using the SFD relation dictionary for clustering, diseases are grouped into 20 categories based on similar rehabilitation measures, as shown in Table 16.

**Table 16:** Suitable rehabilitation measures clustering results based on diseases (Partial).

category	types of diseases	name of the disease	commonly appropriate rehabilitation measures
1	5	Fracture of the right radius and ulna, Fracture of the transverse process of the thoracic vertebra, Ankle sprain, Degenerative arthritis, Osteoarthritis	external application of Chinese herbal medicine or medicinal liquor
2	3	Tendonitis or Tendon strain, Dislocation, Wrist fasciitis	Light and nutritious diet, fresh fruits and vegetables, herbal heat application, local warmth, etc.
3	9	Lower limb nerve injury, Incomplete paralysis, Thigh muscle atrophy, Calf muscle atrophy, Soft tissue injury of the skin, Ankle ligament tear, Ankle joint fracture, Patellar rupture, Patellar fracture	rehabilitation training
4	5	Extensor tendon injury, Synovial lesion or Synovial pathology, Neuropathy or Nerve pathology, Tendinopathy or Tendon pathology, Tenosynovitis	color Doppler ultrasound, MRI
5	10	Joint sprain, Coccyx fracture, Fracture of the proximal phalanx of the fifth toe on the left foot, Comminuted fracture, Muscle atrophy, Fracture of the left L1 transverse process (in the lumbar region), Lumbar spine fracture, Patellar fracture, Cervical vertebra fracture, Femoral neck fracture	bed rest
6	6	Soft tissue injury of the joint, Distal radioulnar joint separation, Sprain, Soft tissue contusion, Soft tissue ligament strain, Rheumatoid arthritis	safflower oil

Table 16 includes the category number, the number of diseases, their names, and suitable rehabilitation measures for each category. clustering based on rehabilitation measures results in 19 categories with 123 diseases that show typical symptom characteristics. the 20th category has 270 diseases but lacks typical rehabilitation measures, leading to low differentiation. For diseases with clear rehabilitation measures, there is significant variation. Categories 4, 6, 8, 11, 12, 13, 15, 16, 17, and 19 involve more specialized treatments like medication or surgery. the remaining categories focus on daily activities, such as exercise and healthy diet. Category 1 and its measures are detailed in Table 17.

**Table 17:** List of suitable rehabilitation measures for the first disease category.

Index	Type	Size	Value
0	str	1	fracture of the right radius and ulna, physical therapy and acupuncture, external application of Chinese herbal medicine, surgery, rehabilitation training, moxibustion
1	str	1	thoracic transverse process fracture, physical therapy, external application of blood-activating herbal medicine, ibuprofen tablets, hot compress, follow-up X-ray
2	str	1	ankle sprain: rest, physical therapy, external application of Chinese herbal medicine
3	str	1	Degenerative arthritis, external application of Chinese herbal medicine, traditional Chinese medicine therapy, external application of herbal liquor, rest, anti-inflammatory and pain-relieving medication, herbal medicine for bruises
4	str	1	osteoarthritis, X-ray, external application of Chinese herbal medicine, traditional Chinese medicine therapy, knee joint replacement, external application of herbal liquor, rest, anti-inflammatory and pain-relieving medication, herbal medicine for bruises

Table 17 shows that “External Application” or “Hot Compress” is included in all appropriate rehabilitation measures for the first disease category. The clustering analysis of rehabilitation measures can help online health community managers create tailored health science columns for various diseases. These columns can be recommended to rehabilitation patients, supporting their health management.

(3) Clustering Results and Analysis of Inappropriate Rehabilitation Measures.

Using the NSFD relationship dictionary, diseases are grouped into six categories based on similar inappropriate rehabilitation measures, as shown in Table 18.

**Table 18:** Clustering results of diseases based on inappropriate rehabilitation measures.

category	number of illnesses	name of the disease	common inappropriate rehabilitation measures
1	12	fracture of the femoral neck, fracture of the proximal phalanx of the fifth toe, fracture of the radial styloid process, pelvic fracture, fifth metatarsal fracture, fibular fracture, lumbar vertebral fracture, patellar fracture, osteoarthritis of the knee joint, calcaneal fracture, ankle joint fracture, and patellar rupture	weight-bearing
2	20	fracture of the middle phalanx of the index finger, bilateral radial bone cracks, right wrist radial bone crack, right clavicle fracture, left radial bone fracture, left anterior cruciate ligament rupture, fracture of the proximal phalanx of the fifth toe of the left foot, left clavicle fracture, fall-related fractures, skin soft tissue injury, skin allergy, fasciitis, gastritis, ankle ligament tear, foot fracture, navicular bone fracture, clavicle fracture, old fracture, femoral neck fracture, bone crack	spicy and irritating foods
3	12	clay-Shoveler’s fracture, fracture of the right radial styloid process, fracture of the distal end of the right radius, contusion of the proximal end of the ulna and radius, left acromion avulsion fracture, left ulnar and radial dislocation, congenital dislocation of the radius, vertebral compression fracture, elbow joint fracture, left transverse process fracture of the lumbar vertebra, greenstick fracture, cervical spine fracture.	surgery
4	19	joint injury, joint dislocation, articular surface injury, medial collateral ligament injury, wound infection, meniscus injury, fracture of the radial head, local soft tissue injury, fracture of the left radial head, fracture of the left radial head of the left arm, fall-related injury, radial head dislocation, muscle injury, knee joint degeneration, soft tissue injury, ligament strain, partial patellar ligament injury, patellar ligament injury, patellar dislocation	excessive and intense exercise
5	9	fracture of the right radial head, torn posterior ligament in the right leg, fracture of the distal end of the ulna and radius, incomplete fracture of the distal end of the left ulna, linear fracture of the radial head of the left elbow, tendon strain, dislocation, wrist fasciitis, chronic injury of the patellar ligament	spicy, cold/raw foods, exposure to cold and dampness
6	101	fracture with avulsion of the right foot, navicular bone fracture of the right foot, comminuted fracture of the olecranon process of the ulna and radius, coccyx fracture, olecranon bursitis, wedge bone fracture, stenosing tenosynovitis, dermatitis, pilonidal sinus, intercondylar eminence fracture, etc.	

Table 18 presents the clustering of diseases based on common unsuitable rehabilitation measures. This analysis identified five categories with typical symptoms, totaling 72 diseases. the 6th category includes 101 diseases but lacks clear unsuitable rehabilitation measures, leading to low differentiation. Some of these diseases are less represented in the online Q&A community. For example, Table 19 lists the diseases in category 1 along with their unsuitable rehabilitation measures.

**Table 19:** List of inappropriate rehabilitation measures for the first disease category.

Index	Type	Size	Value
0	str	1	femoral neck fracture, weight-bearing
1	str	1	fracture of the proximal phalanx of the fifth toe of the left foot, weight-bearing
2	str	1	fracture of the olecranon process, exercise, weight-bearing
3	st	1	pelvic bone fracture, weight-bearing
4	str	1	fracture of the fifth toe, weight-bearing walking
5	str	1	fibular fracture, weight-bearing, weight-bearing walking
6	str	1	lumbar vertebral fracture, weight-bearing
7	str	1	patellar fracture, weight-bearing
8	str	1	osteoarthritis of the knee joint, activity, weight-bearing walking
9	str	1	calcaneal fracture, activity, rotational swinging, weight-bearing walking, weight-bearing, weight-bearing walking - lateral swinging
18	str	1	ankle joint fracture, weight-bearing, weight-bearing on the lower limb
11	str	1	patellar rupture, exertion, weight-bearing walking

Table 19 shows that all unsuitable rehabilitation measures for Category 1 diseases include “weight-bearing.” The results of clustering can help doctors recommend appropriate rehabilitation measures based on patients’ concerns or potential impact on recovery. Sharing this clustering result with online health community managers can help them categorize diseases by similar unsuitable measures. This enables them to inform patients about rehabilitation practices to avoid, helping them mitigate risks and promote recovery.

## Conclusion and future work

Our research collected and cleaned Q&A data from a rehabilitation community, developing a BERT-BiGRU-attention model to extract four relationship types: DS (Disease-Symptom), SFD (Suitable for Disease), NSF (Not Suitable for Disease), and UKN (Unknown). Through optimized hyperparameter tuning on the validation set, the model achieved exceptional performance on the test set (precision: 96.65%, recall: 96.66%, F1-score: 96.63%, AUPR: 93.15%, AUC: 96.67%), surpassing baseline models like BERT-BiGRU and CNN. The success stems from BERT’s contextual embedding capabilities and the attention mechanism’s noise-filtering efficacy, which collectively enable precise identification of rehabilitation relationship patterns in noisy user-generated content. The extracted knowledge was organized into a relationship dictionary, detailing symptom-based, appropriate, and inappropriate rehabilitation measures through clustering. Future enhancements include expert validation, dataset expansion for broader rehabilitation conditions, exploration of additional entities, and domain-specific BERT pre-training to improve accuracy.

Our research transforms fragmented data from raw patient discussions (e.g., online forum posts) into structured medical knowledge, enhancing the rehabilitation knowledge system and promoting knowledge sharing. It also enables doctors and healthcare systems to utilize information more efficiently. For instance, during remote consultations, the system helps distinguish specific symptoms like “elbow deformity” from generalized descriptions such as “pain,” accelerating diagnosis. It blocks dangerous recommendations (e.g., “weightlifting after fractures”) to mitigate legal risks for healthcare platforms. Additionally, it empowers patients with accessible visual guides (e.g., “safe muscle exercises”) for personalized self-managed rehabilitation. These achievements bridge the gap between informal patient experiences and formal medical standards, improving healthcare quality and patient self-management capabilities.

Three key limitations require attention. First, the data used in the study primarily comes from tech-savvy adults aged 20–55, potentially overlooking the rehabilitation needs of older populations, such as osteoporosis care. Second, the system struggles with rare or complex medical cases like “fractures with nerve damage” due to insufficient online discussions available for training. Finally, its reliance on textual patterns may lead to misinterpretations of casual language, such as mistaking phrases like “my arm is dead” (a colloquial expression) for literal medical emergencies. Addressing these issues will improve the technology’s fairness, reliability, and safety for diverse user groups.

To address current limitations, future work should focus on three priorities. First, expand data diversity by collaborating with geriatric/pediatric care providers and low-resource language communities to ensure equitable representation of underrepresented groups (e.g., elderly osteoporosis protocols) while validating extracted knowledge through partnerships with physiotherapists. Second, enhance multimodal integration by combining medical imaging, sensor data (e.g., range-of-motion measurements), and patient-reported outcomes to cross-verify rehabilitation insights and reduce reliance on text-only patterns. Third, improve contextual language understanding using advanced sarcasm/colloquialism detection algorithms and synthetic data generation for rare conditions (e.g., fractures with neuropathy), ensuring safer interpretations of informal patient expressions. These steps aim to build a clinically robust, linguistically aware, and socially inclusive framework for rehabilitation knowledge extraction.

## Data Availability

The data that support the findings of this study are available from the author-Yanli Zhang(zhangyanli_sufe@126.com) upon reasonable request.

## Declarations

### Human and animal participants

This study analyzed only pre-existing, anonymized public forum posts collected through text mining, with no direct interaction with human participants. The research qualifies as ‘not human subjects research’ under 45 CFR 46.104(d)(4), as it involved secondary analysis of non-identifiable, publicly available data. All data were anonymized by the source platform prior to acquisition.
